# Metabolic fitness of *Candida albicans* is indispensable for functional drug efflux, ergosterol, and chitin biosynthesis

**DOI:** 10.18502/cmm.6.3.3980

**Published:** 2020-09

**Authors:** Sandeep Hans, Zeeshan Fatima, Saif Hameed

**Affiliations:** 1 Amity Institute of Biotechnology, Amity University Haryana, Gurugram (Manesar)-122413, India.

**Keywords:** *Candida*, Chitin, Efflux pump, Ergosterol, Glyoxylate cycle

## Abstract

**Background and Purpose:**

The increment in fungal infections, particularly due to *Candida* species, is alarming due to the emergence of multidrug resistance (MDR). Hence, the identification of novel drug targets to circumvent the problem of MDR requires immediate attention. The metabolic pathway, such as glyoxylate cycle (GC), which utilizes key enzymes (isocitrate lyase [ICL] and malate synthase [MLS]), enables *C. albicans* to adapt under glucose-deficient conditions. This study uncovers the effect of GC disruption on the major MDR mechanisms of *C. albicans* as a human pathogenic fungus.

**Materials and Methods:**

For the purpose of the study, efflux pump activity was assessed by phenotypic susceptibilities in the presence of substrates rhodamine 6G (R6G) and Nile red, along with R6G extracellular concentration (527 nm). In addition, ergosterol content was estimated by the alcoholic potassium hydroxide hydrolysis method. The estimation of chitin was also accomplished by the absorbance (520 nm) of glucosamine released by acid hydrolysis.

**Results:**

The results revealed that the disruption of ICL enzyme gene (Δicl1) led to the impairment of the efflux activity of multidrug transporters belonging to the ATP - binding cassette superfamily. It was further shown that Δicl1 mutant exhibited diminished ergosterol and chitin contents. In addition, all abrogated phenotypes could be rescued in the reverting strain of Δicl1 mutant.

**Conclusion:**

Based on the findings, the disruption of GC affected efflux activity and the synthesis of ergosterol and chitin. The present study for the first time revealed that metabolic fitness was associated with functional drug efflux, ergosterol and chitin biosynthesis and validated GC as an antifungal target. However, further studies are needed to comprehend and exploit this therapeutic opportunity.

## Introduction

*Candida albicans* is a prevalent human fungal pathogen and leading cause of nosocomial infections, particularly in individuals with immunocompromised conditions, such as HIV/AIDS, cancer, transplantations, and neonatal infections [ 1 , 2 ]. The impeded progress in the development of new antifungal drugs and concomitant rise in multidrug resistance (MDR) have even worsened the problems associated with such infections [ 3 , 4 ]. Therefore, it is pertinent to identify novel drug targets to circumvent the phenomenon of MDR. The pathogenicity of *C. albicans* mainly depends upon adaptability to combat diverse stress conditions existing inside the hostile niche by the activation of various stress-induced pathways. Regarding this, the in vitro analysis of these pathways and their roles in stress adaptation have generated considerable interest to identify potential antifungal targets.

Although *C. albicans* mainly utilizes six-carbon glucose as the key source of metabolism, they frequently encounter sites with low carbon compounds, such as acetate and citrate, which must be utilized to trigger virulence [ 5 , 6 ]. Therefore, the metabolic fitness of *C. albicans* is a crucial factor, which is required as a strategy to adapt to nutrient-limited conditions and establish a successful infection. Glyoxylate cycle (GC) is one such pathway that operates under metabolic stress and acts as a homeostatic mechanism to adjust various metabolic activities in *C. albicans*. In microorganisms, including *C. albicans*, GC acts as a metabolic shunt for tricarboxylic acid cycle to enable the consumption of the low number of carbon (C_2_) compounds in a two-step process when glucose is not readily present as a carbon source. Therefore, this cycle not only utilizes C_2_ compounds but also prevents the loss of the two carbons, bypassing the CO_2_ generating steps in Krebs cycle [ 7 , 8 ]. The key enzymes of GC include isocitrate lyase (ICL) and malate synthase (MLS) encoded by *ICL1* and *MLS1*, respectively. These enzymatic pathways are absent in humans; therefore, they serve as attractive antifungal targets [ 9 ].

It has been already established that *C. albicans* strains lacking either *ICL1* or *MLS1* are less virulent [ 10 , 11 ]. However, a comprehensive study deciphering the role of GC in other cellular responses responsible for regulating drug resistance in *C. albicans* was still elusive. With this background in mind, the present study was conducted to elucidate the effect of GC disruption on drug efflux pump activity and levels of ergosterol and chitin in *C. albicans*. This is the first study that reports that functional GC is indispensable when one considers dissecting drug resistance mechanisms of *C. albicans*.

## Materials and Methods

**Materials**

Yeast nitrogen base (YNB), agar, Rhodamine 6G (R6G), Nile red (NR), dinitrophenol, and glycerol were purchased from the HiMedia Co. (Mumbai, India). In addition, D-glucose, 2, deoxy-glucose, acetylacetone, SDS, and β-mercaptoethanol were obtained from the CDH Co. (India), and n-Heptane was supplied by the Sigma Chemical Co. (St. Louis, MO, USA).

**Candida strains and media**

*Candida albicans* SC5314 was used as a wild type (WT) strain, along with GC mutants, MRC10 (∆*icl1/icl1*), and MRC11 (∆*icl1*+ICL1**) generated from SC5314 and kindly provided by the Lorenz group [ 12 ]. Δicl1 is a homozygous null mutant of *ICL1*, while Δicl1*+ICL1* is a reconstituted (revertant) strain. All strains were grown in YNB, containing 0.67% YNB and 2% agar (for solid plates), supplemented with 2% glycerol to induce GC.

**Spot assay**

To check phenotypic susceptibility, spot assay was performed as described previously [ 13 ]. Briefly, 5 μl of five-fold serially diluted yeast cultures (cells suspended in normal saline to an OD_600_ of 0.1 nm) was spotted on YNBA plates, supplemented with 2% glycerol in the presence of R6G and NR. Growth difference was measured after incubation for 48 h at 30°C.

**Rhodamine 6G Efflux**

The efflux of R6G was determined as described previously [ 13 ]. Briefly, 1×10^6^ yeast cells from the overnight-grown cultures of WT, Δicl1, and Δicl1+ *ICL1* were transferred to YNB medium, supplemented with 2% glycerol and allowed to grow for 5 h. The cells were then pelleted, washed twice with phosphate- buffered saline (PBS; without glucose), and resuspended as a 2% cell suspension, corresponding to 108 cells in PBS without glucose.

The cells were then de-energized to deplete any ATP for 45 min in 2-DOG (5 mM) and 2,4 DNP (5 mM) in PBS (without glucose). The de-energized cells were pelleted, washed, resuspended as a 2% cell suspension (wt/vol) in PBS without glucose, and then added with R6G at a final concentration of 10 µM and incubated for 40 min at 30°C. Subsequently, the equilibrated cells with R6G were washed and resuspended as a 2% cell suspension (wt/vol) in PBS without glucose. The samples with a volume of 1 ml were withdrawn at the indicated time and centrifuged at 9,000 g for 2 min. The supernatant was then collected, and absorption was measured at 527 nm. Energy-dependent efflux (at the indicated time) was measured after the addition of glucose (2%) to the cells resuspended in PBS (without glucose). Glucose-free negative controls were also included in all experiments.

**Quantitation of ergosterol**

Sterols were extracted by the alcoholic KOH method; additionally, the percentage of ergosterol was calculated as described previously [ 14 ]. Briefly, a single *C. albicans* colony from the overnight cultures of WT, Δicl1, and Δicl1*+ICL1* was used to inoculate 50 mL YNB medium, supplemented with 2% glycerol. Both ergosterol and 24(28)-DHE were absorbed at 281.5 nm, whereas only 24(28)-DHE was absorbed at 230 nm. Ergosterol content was determined by subtracting the amount of 24(28)-DHE (calculated from OD_230_) from the total ergosterol plus 24(28)-DHE content (calculated from OD281.5). Ergosterol content was measured as the percentage of the wet weight of the cells using the following equations:

%ergosterol+%24(28)-DHE=[(A281.5/290)×𝐹]/pellet weight;

% 24(28)-DHE=[(A230/518)×𝐹]/pellet weight

and %ergosterol=[%ergosterol+%24(28) DHE] −% 24(28) DHE

where 𝐹 is the factor for dilution in petroleum ether, and 290 and 518 are the 𝐸-values (in percent per centimeter) determined for crystalline ergosterol and 24(28)-DHE, respectively.

**Chitin estimation**

The chitin content was estimated by measuring the absorbance of glucosamine released by acid hydrolysis as described previously with some modifications [ 15 ]. Briefly, the overnight-grown cultures of WT, Δicl1, and Δicl1+*ICL1* were inoculated in YNB medium supplemented with 2% glycerol, and 1×10^6^ yeast cells were collected by centrifugation. The cells were then washed, suspended in sterile distilled water, and disrupted with 0.5-mm glass beads in a homogenizer. Cell debris was washed five times with 1 M NaCl. The cell wall was extracted in SDS-MerOH extraction buffer (50 Mm Tris, 2% sodium dodecyl sulfate, 0.3 M β-mercaptoethanol, 1 mM EDTA, pH of 8.0) at 100°C for 10 min.

The extracts were washed three times in sterile distilled water and dried at 65°C. Approximately 4 mg dry weight of the cell wall extract was hydrolyzed in 1 mL of 6M HCl at 100°C for 17 h. After evaporation at 65°C, 1 mL of sterile distilled water was added. A volume of 0.1 mL of this sample was mixed with 0.1 mL of 1.5 M Na_2_CO_3_ in 4% acetyl acetone. The mixture was incubated at 100°C for 20 min, and then added with 0.7 mL of 96% ethanol and 0.1 mL of 1.6 g of p-dimethylaminobenzaldehyde in 30 mL of concentrated HCl and 30 mL of ethanol. After 1 h, the absorbance was measured at 520 nm using a spectrophotometer. The glucosamine concentration in each sample was determined from a standard curve of 0-0.3 mg/ml of glucosamine. Subsequently, chitin content was calculated as the percentage of cell wall dry weight.

**Statistical analysis**

All experiments were performed in triplicates (n=3). The results were reported as mean±standard deviation and analyzed using Student’s t-test in which only a p-value of < 0.05 for WT vs. Δicl1 was considered statistically significant.

**Ethical considerations**

Not applicable in this study.

## Results

**Inhibitory effect of glyoxylate cycle disruption against ATP-binding cassette superfamily efflux pumps activity**

**Figure 1:**
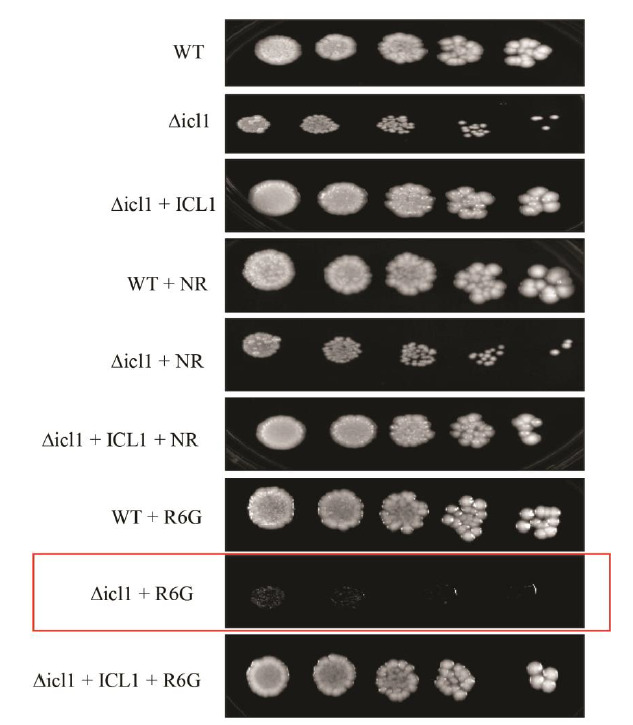
. Effect of *ICL1* disruption on phenotypic susceptibility with R6G and NR (*Candida albicans* strain SC5314 (WT), MRC10 (Δicl1), and MRC11 (Δicl1*+ICL1*) were cultured on YNB agar plates, supplemented with 2% glycerol with or without R6G (10µM) and NR (10 µM) for 2 days at 30°C.)

Initially, the phenotypic susceptibility of WT, Δicl1, and Δicl1*+ICL1* was monitored in the presence of R6G and NR. The results were indicative of the susceptibility of Δicl1 to R6G with no effect on WT and Δicl1*+ICL1* strains. This suggested that the disruption of *ICL1* led to the induction of some inhibitory effects against ABC superfamily transporters functioning as R6G in their specific substrate (Figure 1). Additionally, in order to investigate the probable effects of *ICL1* disruption on major facilitator superfamily (MFS) transporters, the susceptibility of substrate NR was evaluated. Based on the results, Δicl1 was not susceptible to NR, confirming that *ICL1* deletion has no effect on the activity of MFS transporters (Figure 1).

**Figure 2:**
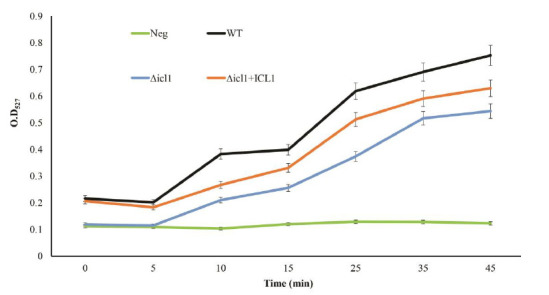
Effect of *ICL1* disruption on R6G efflux (R6G efflux assay was performed using the SC5314 (WT), MRC10 (Δicl1), and MRC11 (Δicl1*+ICL1*) strains of *C. albicans*. The energy-dependent R6G efflux was initiated by adding glucose (2%) and quantified by measuring the absorbance of the supernatant at 527 nm. The values are the means and standard deviations (indicated by error bars] obtained from three independent experiments. Negative control represents efflux without the addition of glucose.)

To further confirm this hypothesis, the functionality of efflux pumps in WT, Δicl1, and Δicl1+ *ICL1* was checked by monitoring R6G efflux, which is a widely used substrate for ABC transporters. To our expectations, the extracellular concentration of R6G was decreased in Δicl1 mutant in comparison to those in WT and Δicl1*+ICL1* strains, confirming the impaired activity of ABC superfamily drug transporters (Figure 2).

**Effect of glyoxylate cycle disruption on reducing ergosterol content**

**Figure 3:**
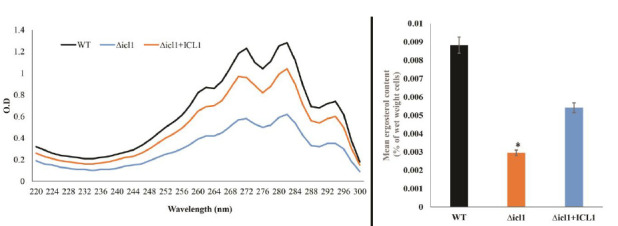
Effect of *ICL1* disruption on ergosterol levels (Left panel depicts the UV spectrophotometric ergosterol profiles of SC5314 (WT), MRC10 (Δicl1), and MRC11 (Δicl1*+ICL1*) strains of *C. albicans* scanned at 220-300 nm. Right panel denotes the relative mean percentage of ergosterol content calculated as described in the method section and normalized by considering the untreated control as 100±SD of three independent sets of experiments depicted on Y-axis, and * represents a *p-value* of < 0.05.)

The perturbation of membrane structure and function in response to GC disruption was also evident from the fact that ergosterol levels, which are targetsfor azole and polyenes, were considerably reduced. Accordingly, *C. albicans* ergosterol biosynthesis was investigated based on the total ergosterol isolated by alcoholic KOH as described previously. The results revealed a significant decline (P<0.05) in ergosterol levels in Δicl1 mutant that could be rescued in the revertant Δicl1+ *ICL1* strain (Figure 3).

**Effect of glyoxylate cycle disruption on reducing chitin content**

**Figure 4:**
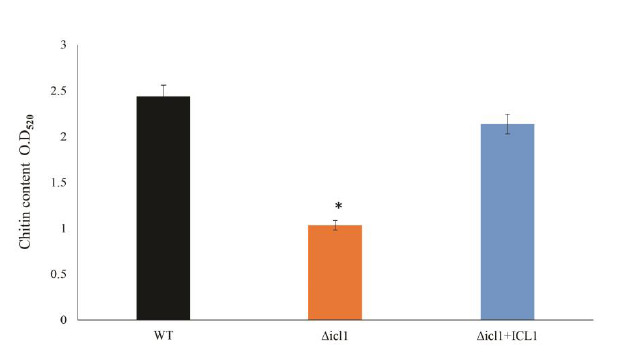
Effect of *ICL1* disruption on chitin levels (Chitin content in SC5314 (WT), MRC10 (Δicl1), and MRC11 (Δicl1*+ICL1*) strains of *C. albicans* are depicted as bar graph and quantified by measuring the absorbance at 520 nm on Y axis, and * denotes a *p-value* of < 0.05.)

This study also involved the examination of the effect of GC disruption on chitin biosynthesis, which is a target for echinocandins. The cell wall is a characteristic structure of fungi; moreover, components of the fungal cell wall are not present in humans; accordingly, this structure is an excellent target for antifungal therapy. To this end, the chitin content in Δicl1 mutant was estimated, and the results revealed the reduction of chitin content as a result of *ICL1* deletion in comparison to those subjected to WT and Δicl1*+ICL1* (revertant) strains (Figure 4). These observations suggested that the disruption of GC also affected cell wall integrity.

## Discussion

Candidiasis is one of the major causes of nosocomial infections with a high mortality rate across the world. Among the various mechanisms, which drive *Candida* to escape from the action of antifungal drugs, the overexpression of drug efflux pumps, as well as alteration in cell membrane and cell wall, are the most dominant strategies for drug resistance. The GC is one of the key cycles, which enables *C. albicans* to survive in a wide variety of glucose-depleted niches to establish candidiasis. Recently, GC cycle has been found to be essential for the virulence of several other pathogens and explored as a potential drug target since it is absent in humans.

In *C. albicans*, MDR is mediated by drug efflux pumps, which belong to either ABC superfamily or MFS [ 16 , 17 ]. The major transporters involved are *Candida* drug resistance proteins, namely CaCdr1p and CaCdr2p, from ABC superfamily, along with CaMdr1p belonging to MFS [ 18 , 19 ]. These results established that blockage in GC would lead to impaired efflux pump activity. In addition, the enhanced susceptibility in the presence of R6G contrary to NR reflected that GC disruption would result in the inhibition of the activity of drug efflux pumps belonging to ABC superfamily (Figure 1). Moreover, the reduced exracellular concentration of R6G in Δicl1 further confirmed the specific inhibition of ABC transporter efflux (Figure 2).

The cell membrane of *C. albicans* is a critical interface that mediates a variety of functions, including sensing and signaling to the external environment [ 20 ]. In this study, the inhibition of efflux pump activity encouraged us to examine the composition of the cell membrane more closely. Hence, we quantified the level of ergosterol, which is one of the main components of the cell membrane and known to regulate drug resistance in *C. albicans* [ 21 ]. The result confirmed that GC disruption leads to perturbation in the cell membrane homeostasis. Moreover, the reduction of ergosterol level in Δicl1 could be one of the reasons for the impaired efflux pump activity observed while considering the fact that ABC transporters display different lipid specificities and are targeted to membrane rafts rich in ergosterol [ 21 , 22 ], which gets depleted in Δicl1 mutant.

The disruption of membrane homeostasis further prompted us to study cell wall, which is another crucial target for drugs belonging to the class of echinocandins, administered against *C. albicans* [ 23 , 24 ]. The cell wall is an essential component in fungal homeostasis. The lack of a covering wall in human cells makes this component an attractive target for antifungal development. Furthermore, elevated chitin content is associated with reduced susceptibility of *Candida* species to caspofungin. Given the observation of a decrease in chitin content in Δicl1 mutant in the present study, it was hypothesized that the disruption of GC also affected chitin biosynthesis.

## Conclusion

Considering the fact that drug efflux is the major MDR mechanism and that ergosterol and chitin biosynthesis are the targets for antifungal azoles and echinocandins, the crosstalks exposed in this study were only fitting. Taken together, the data presented in the current study revealed the indispensability of GC for functional drug efflux, as well as membrane and cell wall integrity in *C. albicans*. However, the mechanisms that underlie this crosstalk remain to be elucidated. Based on these findings, it is required to design the pharmacological inhibitors of GC to aid in combating MDR. The identification of these mechanisms may hold promise for revealing new therapeutic targets for the treatment of life-threatening fungal diseases.
